# Supplementary material for: A robust and efficient method for Mendelian randomization with hundreds of genetic variants
Source: Nat Commun. 2020 Jan 17;11:376. doi: 10.1038/s41467-019-14156-4 (PMC6969055; doi:10.1038/s41467-019-14156-4)
Supplement: Supplementary file 2 — Supplementary Information New [file 41467_2019_14156_MOESM2_ESM.pdf]

Supplementary Information

Supplementary Figures

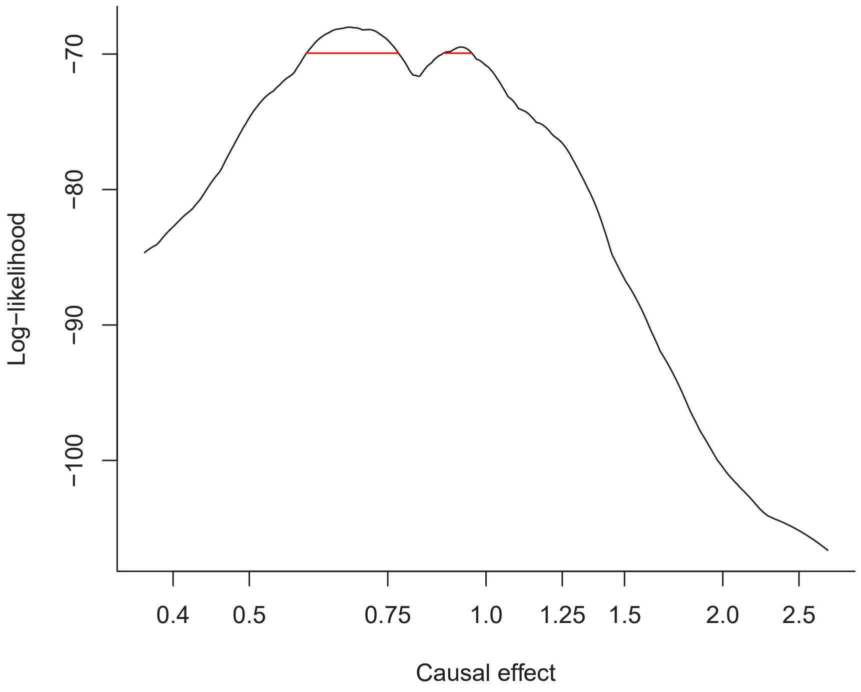

Supplementary Figure 1: Log-likelihood function for causal effect of HDL-cholesterol on CHD risk with recommended initial value of standard deviation 1.114. Red line indicates the 95% confidence interval for the causal effect. Causal estimate represents odds ratio for coronary heart disease per 1 standard deviation increase in HDL-cholesterol.

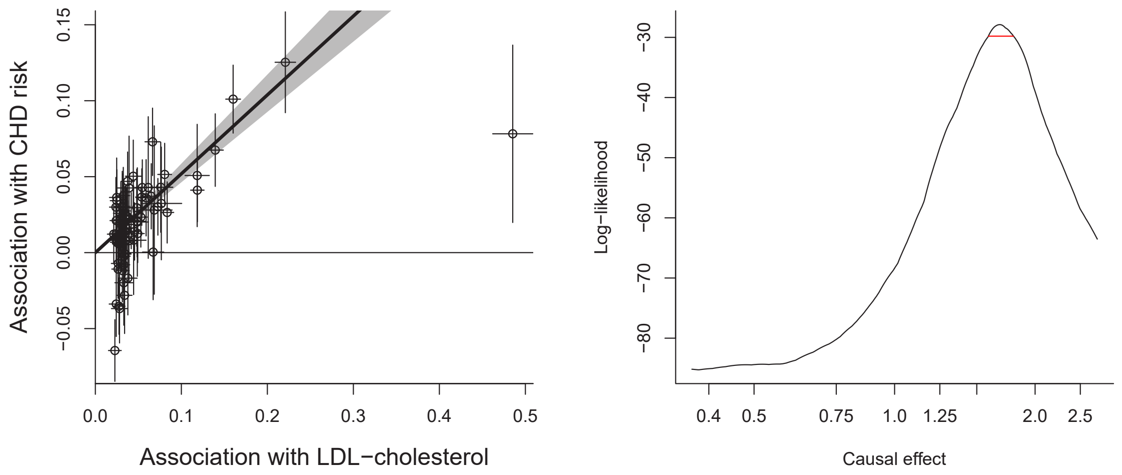

Supplementary Figure 2: Left panel: Genetic associations with LDL-cholesterol (standard deviation units) against genetic associations with CHD risk (log odds ratios). Error bars for genetic associations are 95% confidence intervals. Heavy black line is the causal estimate from the contamination mixture method. The grey area is the 95% confidence interval for the causal effect.

Right panel: Log-likelihood function for causal effect of LDL-cholesterol on CHD risk with recommended initial value of standard deviation 1.008. Red line indicates the 95% confidence interval for the causal effect. Causal estimate represents odds ratio for coronary heart disease per 1 standard deviation increase in HDL-cholesterol.

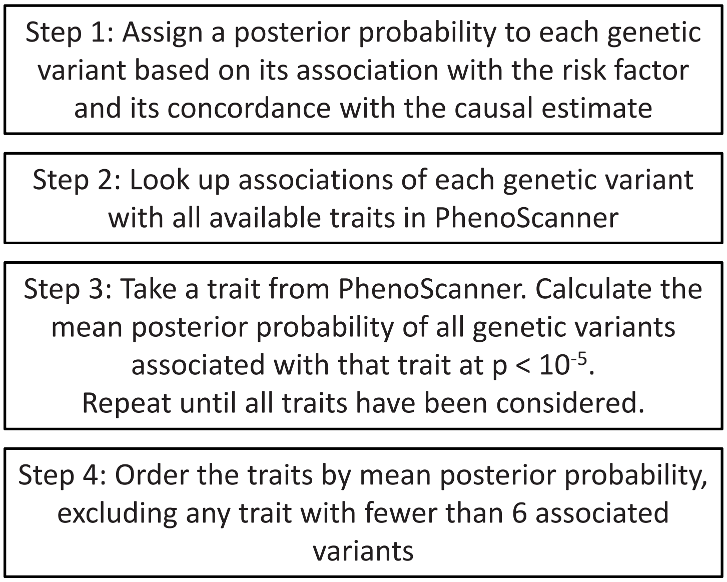

Supplementary Figure 3: Flowchart illustrating approach for searching and ranking risk factors with available summarized data.

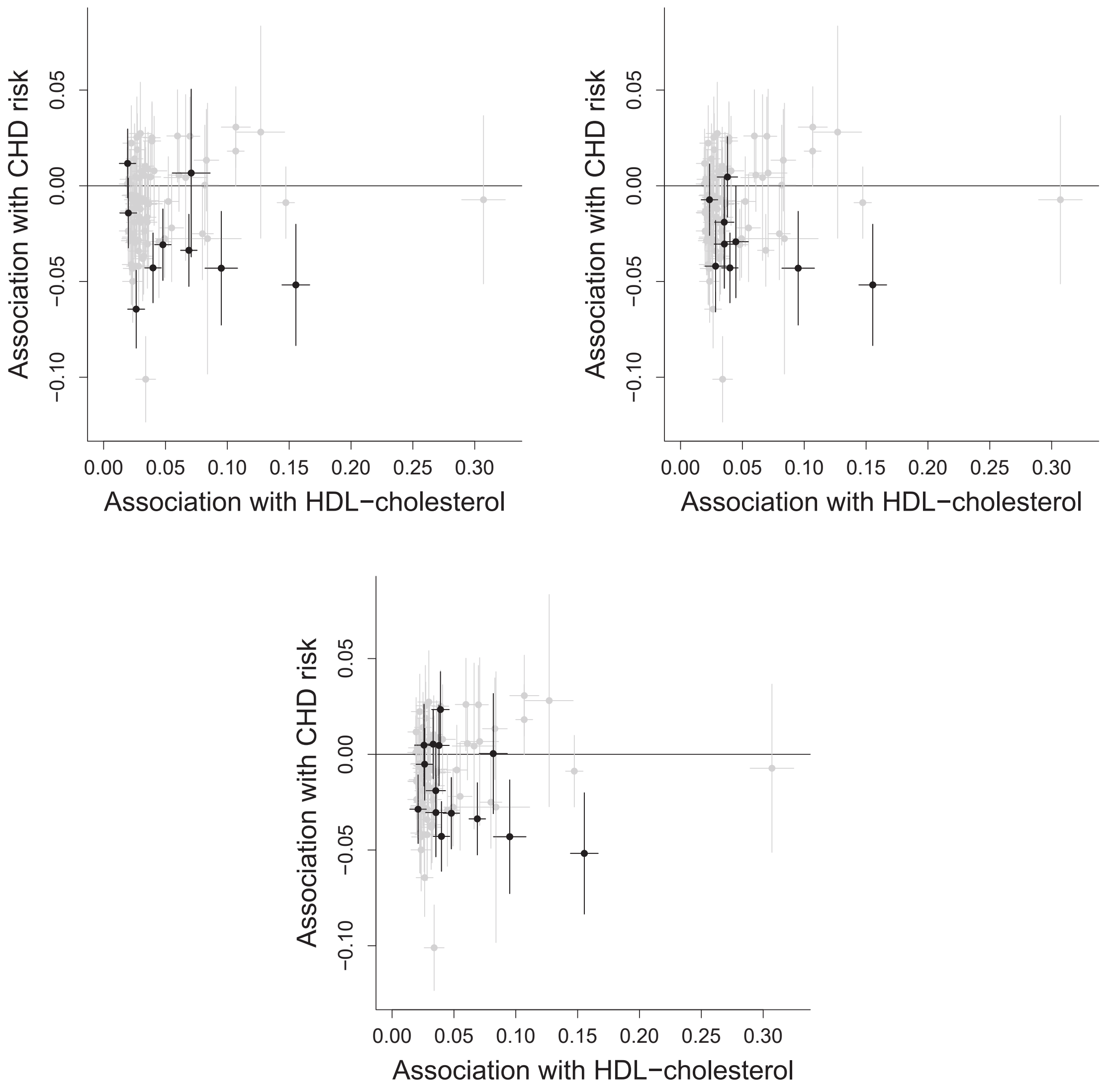

Supplementary Figure 4: Genetic associations with HDL-cholesterol (standard deviation units) against genetic associations with CHD risk (log odds ratios). Variants in black are associated at a suggestive level of significance (p-value $< 10^{-5}$) with: (top left) platelet distribution width, (top right) mean corpuscular hemoglobin concentration, and (bottom) red cell distribution width.

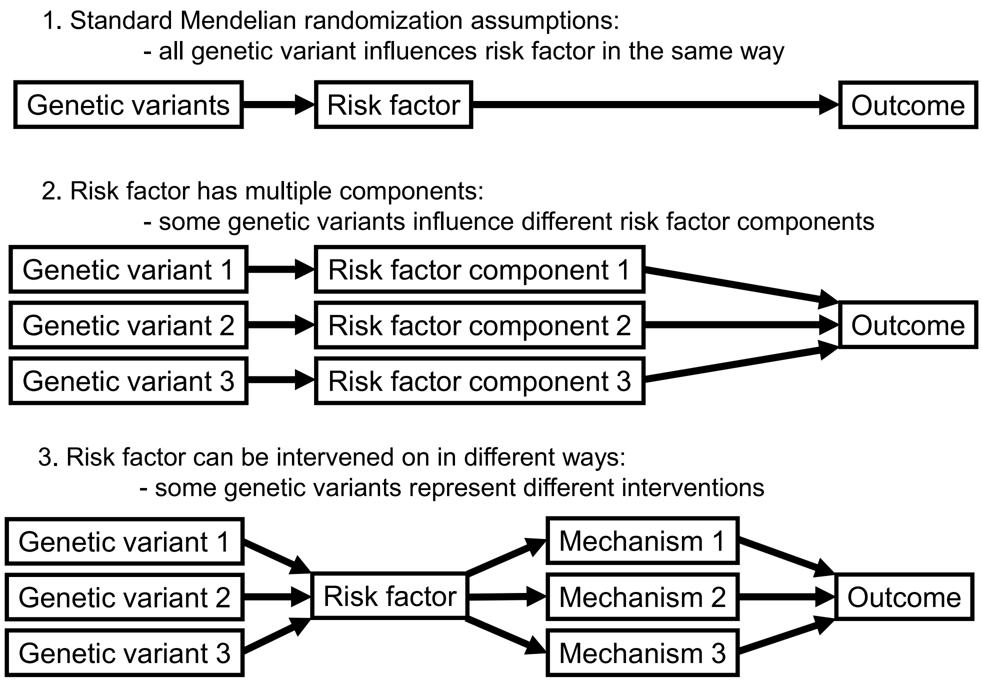

Supplementary Figure 5: Schematic diagram indicating how multiple causal effects could be evidenced for a single risk factor. Some of these pathways may operate via alternative risk factors, which may represent mediators of the association between the risk factor and outcome, or precursors of the risk factor.

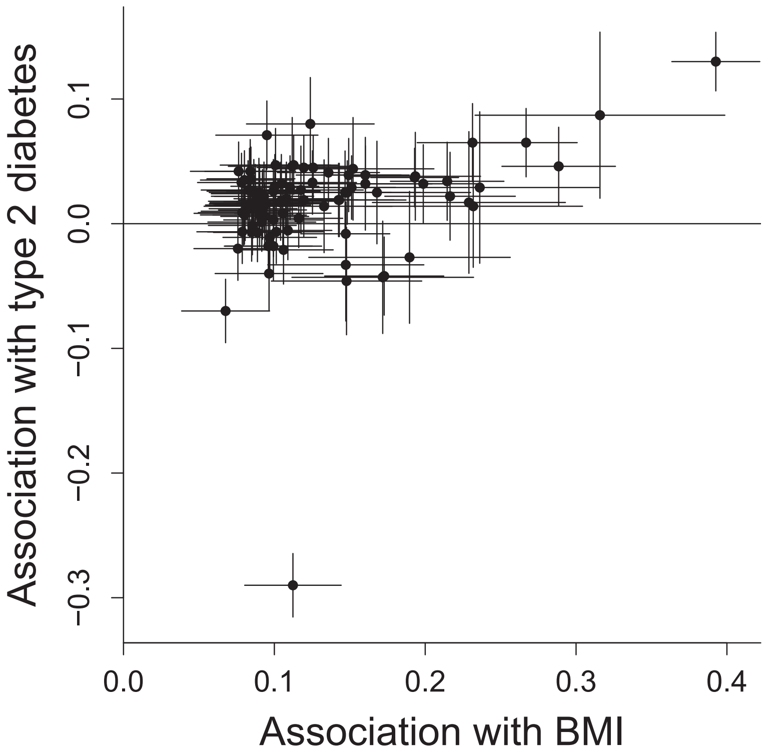

Supplementary Figure 6: Genetic associations with body mass index (BMI, kg/m^2) against genetic associations with type 2 diabetes risk (log odds ratios).

Supplementary Tables

δ_X	δ_Y	Null causal estimate ($\theta = 0$)		Positive causal estimate ($\theta = +0.1$)	
		Mean estimate	Coverage	Mean estimate	Coverage
+1	+1	-0.002	95.3	0.132	93.1
-1	+1	0.004	93.3	0.132	92.9
+1	-1	-0.001	95.8	0.130	92.8
-1	-1	0.001	94.7	0.133	92.9

Supplementary Table 1: Additional simulation to investigate bias and coverage (%) of contamination mixture method when varying effects of the confounder on the risk factor (δ_X) and on the outcome (δ_Y) in Scenario 1 with only 10 genetic variants (all valid instruments) and a sample size of 5000 for the genetic associations. Coverage is the proportion of confidence intervals containing the true causal parameter, and should be should be close to 95% in all cases.

	Number of variants invalid:								
	20	40	60	20	40	60	20	40	60
Method	Scenario 2			Scenario 3			Scenario 4		
Inverse-variance weighted	95.0	94.5	94.3	6.2	0.0	0.0	18.9	0.8	0.0
MR-Egger	93.9	94.5	94.2	93.5	94.3	93.5	24.2	5.6	1.1
Weighted median	95.4	92.1	86.5	81.4	16.7	0.2	51.8	3.4	0.0
MR-PRESSO	91.3	87.9	80.5	99.5	98.7	86.7	99.5	86.4	69.2
Weighted MBE	99.2	97.9	96.7	99.5	98.7	86.7	99.5	86.4	69.2
Contamination mixture	93.1	90.2	83.6	90.8	74.1	34.1	90.3	78.0	38.7

Supplementary Table 2: Coverage of 95% confidence interval for positive causal effect ($\theta = +0.1$).

rsid	Nearest gene	Effect allele	Association with HDL-c Beta (SE)	Association with TG Beta (SE)	Association with CHD risk Beta (SE)	Association with... MCHC	PDW	RCDW
rs4660293	*PABPC4*	A	0.035 (0.004)	-0.020 (0.004)	-0.019 (0.012)	✓		✓
rs12133576	*DR1*	A	0.024 (0.004)	-0.009 (0.003)	-0.006 (0.010)			
rs646776	*CELSR2*	C	0.034 (0.004)	-0.003 (0.004)	-0.101 (0.011)			
rs12145743	*RRNAD1*	G	0.020 (0.004)	-0.012 (0.004)	0.003 (0.010)			
rs4650994	*C1orf220*	G	0.021 (0.003)	-0.002 (0.003)	-0.029 (0.009)			✓
rs1689797	*LINC01344*	C	0.036 (0.004)	-0.011 (0.004)	-0.010 (0.010)			
rs2642438	*MARCH1*	G	0.030 (0.004)	-0.017 (0.004)	0.008 (0.011)			
rs4846914	*GALNT2*	A	0.048 (0.003)	-0.040 (0.003)	-0.031 (0.010)		✓	✓
rs1367117	*APOB*	G	0.022 (0.004)	-0.025 (0.004)	-0.041 (0.011)			
rs7607980	*COBLL1*	C	0.045 (0.005)	-0.036 (0.005)	-0.029 (0.015)	✓		
rs7422339	*CPS1*	C	0.027 (0.004)	0.000 (0.004)	0.025 (0.011)			
rs1515110	*NEU2*	G	0.032 (0.004)	-0.026 (0.003)	-0.037 (0.010)			
rs2290547	*SETD2*	C	0.030 (0.005)	-0.010 (0.004)	0.027 (0.014)			
rs2240327	*RBM6*	G	0.024 (0.003)	-0.002 (0.003)	-0.025 (0.010)			
rs13326165	*STAB1*	A	0.029 (0.004)	-0.020 (0.004)	-0.016 (0.013)			
rs6805251	*GSK3B*	T	0.020 (0.004)	-0.001 (0.003)	-0.014 (0.009)		✓	
rs687339	*KRT18P35*	C	0.032 (0.004)	-0.029 (0.004)	-0.038 (0.011)			
rs10019888	*snoU13*	A	0.027 (0.005)	-0.023 (0.004)	-0.006 (0.013)			
rs442177	*AFF1*	G	0.022 (0.003)	-0.031 (0.003)	0.001 (0.009)			
rs3822072	*FAM13A*	G	0.025 (0.003)	-0.018 (0.003)	-0.019 (0.009)			
rs2602836	*ADH5*	A	0.019 (0.003)	-0.009 (0.003)	0.001 (0.010)			
rs13107325	*SLC39A8*	C	0.071 (0.008)	-0.031 (0.008)	0.007 (0.022)		✓	
rs6450176	*ARL15*	G	0.025 (0.004)	-0.019 (0.004)	-0.030 (0.010)			
rs9686661	*C5orf67*	C	0.028 (0.004)	-0.038 (0.004)	-0.042 (0.012)	✓		
rs205262	*C6orf106*	A	0.028 (0.004)	-0.003 (0.004)	-0.034 (0.011)			
rs998584	*VEGFA*	C	0.026 (0.004)	-0.029 (0.004)	-0.042 (0.010)			
rs9491696	*RSPO3*	C	0.020 (0.003)	-0.018 (0.003)	-0.013 (0.009)			
rs634869	*AL356739.1*	C	0.023 (0.003)	-0.027 (0.003)	-0.007 (0.010)	✓		
rs702485	*DAGLB*	G	0.024 (0.003)	-0.002 (0.003)	-0.012 (0.010)			
rs10282707	*SNX13*	C	0.025 (0.004)	-0.009 (0.003)	0.014 (0.009)			
rs4917014	*AC020743.3*	G	0.022 (0.004)	-0.001 (0.004)	-0.027 (0.010)			
rs17145738	*TBL2*	T	0.041 (0.005)	-0.115 (0.005)	0.008 (0.015)			
rs3996352	*KLF14*	G	0.030 (0.003)	-0.018 (0.003)	-0.019 (0.009)			
rs17173637	*AOC1*	T	0.036 (0.006)	-0.021 (0.006)	-0.009 (0.017)			
rs4240624	*RP11-115J16.1*	A	0.082 (0.006)	-0.028 (0.006)	0.000 (0.016)			✓
rs12678919	*LPL*	G	0.155 (0.006)	-0.170 (0.006)	-0.052 (0.016)	✓	✓	✓
rs894210	*LPL*	A	0.069 (0.003)	-0.067 (0.003)	-0.034 (0.010)		✓	✓
rs2293889	*TRPS1*	G	0.031 (0.004)	-0.006 (0.003)	-0.007 (0.010)			
rs2980885	*RP11-136O12.2*	A	0.035 (0.004)	-0.058 (0.004)	-0.030 (0.012)	✓		✓
rs2954022	*RP11-136O12.2*	A	0.040 (0.003)	-0.078 (0.003)	-0.043 (0.009)	✓	✓	✓
rs4075205	*GPIHBP1*	T	0.022 (0.004)	-0.009 (0.003)	0.022 (0.010)			
rs686030	*TTC39B*	A	0.055 (0.005)	0.025 (0.005)	-0.022 (0.014)			
rs1883025	*ABCA1*	C	0.070 (0.004)	0.022 (0.004)	0.026 (0.010)			
rs2472509	*ABCA1*	G	0.023 (0.004)	-0.002 (0.004)	-0.005 (0.010)			
rs970548	*MARCH8*	C	0.026 (0.004)	0.002 (0.004)	0.005 (0.011)			✓
rs7897379	*REEP3*	C	0.019 (0.003)	-0.027 (0.003)	0.012 (0.009)		✓	
rs2255141	*GPAM*	A	0.034 (0.004)	-0.021 (0.004)	0.010 (0.010)			
rs326214	*MADD*	G	0.061 (0.004)	-0.024 (0.004)	0.006 (0.010)			
rs17788930	*FNBP4*	A	0.036 (0.004)	-0.011 (0.004)	0.009 (0.010)			
rs11246602	*OR4C46*	C	0.034 (0.005)	-0.009 (0.005)	-0.008 (0.014)			
rs12226802	*OR4C15*	G	0.033 (0.005)	-0.007 (0.005)	-0.008 (0.014)			
rs1535	*FADS2*	A	0.039 (0.004)	-0.046 (0.004)	0.023 (0.010)			✓
rs12801636	*PCNX3*	A	0.024 (0.004)	-0.018 (0.004)	-0.050 (0.011)			
rs499974	*RN7SL786P*	C	0.026 (0.004)	0.009 (0.004)	-0.009 (0.012)			
rs10790162	*BUD13*	G	0.095 (0.007)	-0.230 (0.006)	-0.043 (0.015)	✓	✓	✓
rs7117842	*UBASH3B*	C	0.027 (0.004)	-0.002 (0.003)	0.019 (0.010)			
rs11045163	*RP11-284H19.1*	G	0.022 (0.004)	-0.010 (0.003)	-0.005 (0.010)			
rs3741414	*INHBC*	T	0.030 (0.004)	-0.028 (0.004)	-0.012 (0.012)			
rs2241210	*UBE3B*	G	0.033 (0.004)	0.003 (0.003)	0.005 (0.009)			✓
rs653178	*ATXN2*	T	0.026 (0.004)	-0.010 (0.003)	-0.064 (0.010)		✓	
rs838876	*SCARB1*	A	0.049 (0.004)	-0.005 (0.004)	-0.028 (0.010)			
rs10773105	*SCARB1*	C	0.036 (0.004)	-0.004 (0.003)	-0.009 (0.009)			
rs4983559	*RP11-477I4.4*	G	0.020 (0.004)	0.000 (0.004)	-0.014 (0.010)			
rs2412710	*CAPN3*	G	0.084 (0.014)	-0.099 (0.013)	-0.028 (0.036)			
rs492571	*FRMD5*	T	0.066 (0.009)	-0.080 (0.009)	0.004 (0.022)			
rs1532085	*LOC101928635*	A	0.107 (0.004)	0.031 (0.003)	0.018 (0.009)			
rs261342	*LIPC*	G	0.107 (0.006)	0.045 (0.006)	0.031 (0.011)			
rs2652834	*LACTB*	G	0.028 (0.004)	-0.025 (0.004)	-0.006 (0.012)			
rs9930333	*FTO*	T	0.020 (0.004)	-0.021 (0.004)	-0.024 (0.009)			
rs9989419	*AC012181.1*	G	0.147 (0.004)	-0.024 (0.004)	-0.009 (0.010)			
rs5880	*CETP*	G	0.307 (0.009)	-0.048 (0.008)	-0.007 (0.022)			
rs16942887	*PSKH1*	A	0.083 (0.005)	-0.012 (0.005)	0.013 (0.014)			
rs2925979	*CMIP*	C	0.035 (0.004)	-0.020 (0.004)	-0.016 (0.010)			
rs931992	*TCAP*	T	0.034 (0.004)	-0.008 (0.004)	-0.018 (0.010)			
rs4148005	*ABCA8*	T	0.028 (0.004)	-0.007 (0.004)	-0.029 (0.010)			
rs4969178	*PGS1*	G	0.026 (0.004)	-0.018 (0.003)	-0.005 (0.010)			✓
rs4939883	*SMUG1P1*	C	0.080 (0.004)	0.005 (0.004)	-0.025 (0.012)			
rs11660468	*SMUG1P1*	T	0.039 (0.003)	-0.001 (0.003)	0.025 (0.010)			
rs952044	*RP11-795H16.3*	C	0.023 (0.004)	-0.010 (0.004)	-0.043 (0.010)			
rs2278236	*ANGPTL4*	A	0.033 (0.004)	-0.014 (0.003)	-0.024 (0.010)			
rs731839	*PEPD*	A	0.022 (0.004)	-0.022 (0.004)	-0.028 (0.010)			
rs17695224	*FPR3*	G	0.029 (0.004)	-0.012 (0.004)	0.004 (0.011)			
rs103294	*AC010518.3*	T	0.052 (0.004)	-0.002 (0.004)	-0.008 (0.012)			
rs1800961	*HNF4A*	C	0.127 (0.010)	0.002 (0.009)	0.028 (0.028)			
rs4465830	*ZNF335*	A	0.060 (0.004)	-0.053 (0.004)	0.026 (0.012)			
rs181362	*UBE2L3*	C	0.038 (0.004)	0.010 (0.004)	0.005 (0.011)	✓		✓

Supplementary Table 3: Details of genetic variants, beta-coefficients (standard errors, SE) for associations with HDL-cholesterol and triglycerides (TG) (both instandard deviation units) and with coronary heart disease (CHD) risk (log odds ratios), for 86 genetic variants. Tickmarks indicate associations at $p < 10^{-5}$ with mean corpuscular hemoglobin concentration (MCHC), platelet distribution width (PDW), or red cell distribution width (RCDW).

Risk factor	Data source	Mean posterior probability
Platelet distribution width	27863252	0.1868
Mean corpuscular hemoglobin concentration	27863252	0.1631
Red cell distribution width	27863252	0.1385
Coronary artery disease	26343387	0.1048
Platelet count	27863252	0.0798
Reticulocyte fraction of red cells	27863252	0.0674
Waist hip ratio adjusted for BMI	25673412	0.0631
Total cholesterol levels	28334899	0.0587
Plateletcrit	27863252	0.0562

Supplementary Table 4: List of top risk factors from PhenoScanner ranked by mean posterior probability for applied example. Data source is the PubMed ID of the manuscript from which the association estimates were obtained.

Gene	Colocalization group 1	Colocalization group 2	Conservative	Uniform
C1orf220	HDL-c, (CHD), MCHC, RCDW	-		✓
GALNT2	HDL-c, TG, (CHD), PDW, RCDW	-		✓
COBLL1	HDL-c, TG, MCHC, RCDW	-		
C5orf67	HDL-c, TG, CHD, MCHC	-	✓	✓
TRIB1	HDL-c, TG, CHD, MCHC, PDW, RCDW	-	✓	✓
LPL	(HDL-c), CHD, PDW	TG, MCHC, RCDW		✓
BUD13	HDL-c, CHD, MCHC, PDW, RCDW	-	✓	✓
ATXN2	HDL-c, CHD	(MCHC), PDW, RCDW		
CMIP	HDL-c, TG, (CHD), (MCHC)	-		✓

Supplementary Table 5: Results from colocalization analysis using conservative and uniform settings for priors. Traits in parentheses only colocalized using uniform prior. For most gene regions, one set of traits that colocalized was identified; for *LPL* and *ATXN2*, two sets were identified. A checkmark indicates that colocalization was observed for the indicated choice of prior between HDL-cholesterol, CHD risk, and at least one of the blood cell traits.
Abbreviations: HDL-c = HDL-cholesterol, TG = triglycerides, CHD = coronary heart disease risk, MCHC = mean corpuscular hemoglobin concentration, PDW = platelet distribution width, RCDW = red cell distribution width.

	Estimate for HDL-cholesterol	95% confidence interval
No adjustment	-0.584	-0.828, -0.340
Adjustment for MCHC	-0.238	-0.651, 0.175
Adjustment for PDW	-0.381	-0.931, 0.169
Adjustment for RCDW	-0.275	-0.759, 0.210
Adjustment for SBP	-0.477	-0.646, -0.307
Adjustment for BMI	-0.487	-0.695, -0.279
Adjustment for T2D	-0.545	-0.888, -0.201

Supplementary Table 6: Estimates of effect of HDL-cholesterol from mediation analysis using 11 variants from negative cluster only: univariable estimate (no adjustment) and multivariable estimates (adjustment for blood cell traits in turn). Estimates represents log odds ratios per 1 standard deviation increase in HDL-cholesterol. Associations attenuated substantially on adjustment for the potential mediators. In contrast, associations did not attenuate on adjustment for alternative cardiovascular risk factors: body mass index (BMI, genetic associations estimated in the GIANT consortium 2015 data release), systolic blood pressure (SBP, genetic associations estimated in the UK Biobank, 2019 Ben Neale data release), or Type 2 diabetes (T2D, genetic associations estimated in the DIAGRAM consortium 2017 data release).

Standard deviation parameter	Estimate	95% confidence interval
$\psi = 0.8$	-0.456	-0.609, -0.332
$\psi = 1.0$	-0.422	-0.535, -0.275
$\psi = 1.2$	-0.400	-0.517, -0.251 and -0.154, -0.030
$\psi = 1.5$	-0.327	-0.478, -0.245 and -0.193, -0.005
$\psi = 1.8$	-0.134	-0.470, 0.003

Supplementary Table 7: Estimates and 95% confidence intervals from contamination mixture method for different values of the standard deviation parameter ψ. Estimates represent log odds ratios for coronary heart disease per 1 standard deviation increase in HDL-cholesterol. In some cases, the 95% confidence interval consists of two disjoint intervals.

	20 invalid variants			40 invalid variants			60 invalid variants		
Method	Mean	SD	Type 1	Mean	SD	Type 1	Mean	SD	Power
Scenario 2: Balanced pleiotropy, InSIDE satisfied									
Original version	0.001	0.033	6.5	0.001	0.043	9.4	0.001	0.065	15.6
Joint maximization	0.005	0.080	7.9	0.002	0.060	9.9	0.002	0.070	16.2
Scenario 3: Directional pleiotropy, InSIDE satisfied									
Original version	0.015	0.033	8.8	0.041	0.045	26.4	0.139	0.164	61.7
Joint maximization	0.606	0.418	72.5	0.740	0.377	87.4	0.770	0.362	95.6
Scenario 4: Pleiotropy via confounder, InSIDE violated									
Original version	0.011	0.035	9.2	0.034	0.055	18.8	0.161	0.159	52.4
Joint maximization	0.113	0.204	29.3	0.079	0.144	33.0	0.155	0.138	61.9

Supplementary Table 8: Comparison of two versions of contamination mixture method: original version of the method in which the standard deviation parameter ψ is treated as known, and joint maximization version in which the likelihood is jointly maximized across both the causal parameter θ and the standard deviation parameter ψ. Simulations were conducted with a null causal effect in the three invalid variant scenarios. Estimates are summarized across 1000 simulated datasets per scenario.

δ_X	δ_Y	Scenario 1			Scenario 2			Scenario 3			Scenario 4		
		Mean	SD	Power	Mean	SD	Power	Mean	SD	Power	Mean	SD	Power
Null causal effect: $\theta = 0$													
1	1	-0.000	0.028	4.8	0.000	0.042	8.4	0.040	0.043	23.7	0.034	0.056	20.2
1	0.5	-0.001	0.021	6.2	-0.001	0.032	8.6	0.028	0.032	21.2	0.068	0.047	58.0
1	-0.5	0.000	0.021	5.6	0.003	0.032	8.4	0.028	0.032	20.3	-0.064	0.047	55.6
0.5	1	-0.001	0.027	5.8	-0.000	0.045	10.5	0.046	0.045	26.5	0.016	0.048	14.8
-0.5	1	-0.000	0.029	5.9	-0.000	0.044	9.6	0.045	0.045	27.4	-0.016	0.046	13.3
Positive causal effect: $\theta = +0.1$													
1	1	0.094	0.030	90.4	0.099	0.046	69.9	0.144	0.051	93.9	0.144	0.076	85.9
1	0.5	0.095	0.022	98.8	0.097	0.034	88.4	0.126	0.037	97.9	0.168	0.050	99.3
1	-0.5	0.096	0.021	99.5	0.094	0.031	91.4	0.123	0.031	99.6	0.032	0.045	40.2
0.5	1	0.099	0.029	93.6	0.100	0.046	72.8	0.145	0.048	95.2	0.116	0.048	83.2
-0.5	1	0.097	0.028	95.0	0.100	0.046	76.8	0.144	0.046	97.1	0.085	0.044	67.4

Supplementary Table 9: Additional simulation to investigate performance of contamination mixture method when varying effects of the confounder on the risk factor (δ_X) and on the outcome (δ_Y) in Scenarios 2 to 4 with 40 invalid instrumental variables.

Invalid variants	Scenario 2			Scenario 3			Scenario 4		
	Mean	SD	Power	Mean	SD	Power	Mean	SD	Power
Null causal effect: $\theta = 0$									
20 invalid	-0.001	0.035	7.2	0.015	0.033	8.9	0.010	0.036	9.5
40 invalid	0.001	0.043	9.0	0.043	0.045	24.5	0.032	0.058	19.2
60 invalid	-0.002	0.067	16.4	0.162	0.193	62.6	0.162	0.156	53.1
Positive causal effect: $\theta = +0.1$									
20 invalid	0.095	0.036	81.2	0.113	0.038	92.0	0.108	0.038	89.0
40 invalid	0.095	0.047	66.8	0.147	0.052	94.4	0.145	0.073	89.4
60 invalid	0.103	0.071	58.3	0.301	0.226	97.6	0.302	0.162	92.2

Supplementary Table 10: Additional simulation to investigate performance of contamination mixture method when variants are drawn from a normal distribution in Scenarios 2 to 4.
