# Supplementary material for: A robust and efficient method for Mendelian randomization with hundreds of genetic variants
Source: Nat Commun. 2020 Jan 17;11:376. doi: 10.1038/s41467-019-14156-4 (PMC6969055; doi:10.1038/s41467-019-14156-4)
Supplement: Supplementary file 3 — Description of Additional Supplementary Files [file 41467_2019_14156_MOESM3_ESM.pdf]

Title: Supplementary Software 1
Description: R software code to implement contamination mixture method.

MRC Biostatistics Unit, Cambridge Institute of Public Health
Robinson Way, Cambridge, CB2 0SR

Telephone: 01223 768259
E-mail: sb452@medschl.cam.ac.uk
