# Supplementary material for: A robust and efficient method for Mendelian randomization with hundreds of genetic variants
Source: Nat Commun. 2020 Jan 17;11:376. doi: 10.1038/s41467-019-14156-4 (PMC6969055; doi:10.1038/s41467-019-14156-4)
Supplement: Supplementary file 5 — Reporting Summary [file 41467_2019_14156_MOESM5_ESM.pdf]

nature research

Corresponding author(s): Stephen Burgess

Last updated by author(s): Mar 22, 2019

Reporting Summary

Nature Research wishes to improve the reproducibility of the work that we publish. This form provides structure for consistency and transparency in reporting. For further information on Nature Research policies, see Authors & Referees and the Editorial Policy Checklist .

Statistics

For all statistical analyses, confirm that the following items are present in the figure legend, table legend, main text, or Methods section.

n/a	Confirmed	
☐	☒	The exact sample size (*n*) for each experimental group/condition, given as a discrete number and unit of measurement
☐	☒	A statement on whether measurements were taken from distinct samples or whether the same sample was measured repeatedly
☐	☒	The statistical test(s) used AND whether they are one- or two-sided<br>*Only common tests should be described solely by name; describe more complex techniques in the Methods section.*
☐	☒	A description of all covariates tested
☐	☒	A description of any assumptions or corrections, such as tests of normality and adjustment for multiple comparisons
☐	☒	A full description of the statistical parameters including central tendency (e.g. means) or other basic estimates (e.g. regression coefficient) AND variation (e.g. standard deviation) or associated estimates of uncertainty (e.g. confidence intervals)
☒	☐	For null hypothesis testing, the test statistic (e.g. *F*, *t*, *r*) with confidence intervals, effect sizes, degrees of freedom and *P* value noted<br>*Give P values as exact values whenever suitable.*
☒	☐	For Bayesian analysis, information on the choice of priors and Markov chain Monte Carlo settings
☒	☐	For hierarchical and complex designs, identification of the appropriate level for tests and full reporting of outcomes
☐	☒	Estimates of effect sizes (e.g. Cohen's *d*, Pearson's *r*), indicating how they were calculated

Our web collection on statistics for biologists contains articles on many of the points above.

Software and code

Policy information about availability of computer code

Data collection	This manuscript does not contain primary data, only secondary data from published genome-wide association studies.
Data analysis	All data analysis was performed using R version 3.5.1 ("Feather Spray")

For manuscripts utilizing custom algorithms or software that are central to the research but not yet described in published literature, software must be made available to editors/reviewers. We strongly encourage code deposition in a community repository (e.g. GitHub). See the Nature Research guidelines for submitting code & software for further information.

Data

Policy information about availability of data

All manuscripts must include a data availability statement. This statement should provide the following information, where applicable:

- Accession codes, unique identifiers, or web links for publicly available datasets
- A list of figures that have associated raw data
- A description of any restrictions on data availability

All data used in this manuscript are publicly available and can be downloaded from www.phenoscanner.medschl.cam.ac.uk.

Field-specific reporting

Please select the one below that is the best fit for your research. If you are not sure, read the appropriate sections before making your selection.

☒ Life sciences ☐ Behavioural & social sciences ☐ Ecological, evolutionary & environmental sciences

For a reference copy of the document with all sections, see nature.com/documents/nr-reporting-summary-flat.pdf

Life sciences study design

All studies must disclose on these points even when the disclosure is negative.

Sample size	This manuscript does not contain primary data, only secondary data from published genome-wide association studies. Sample size was the totality of the data available to us.
Data exclusions	This manuscript does not contain primary data, only secondary data from published genome-wide association studies. No data were excluded by us.
Replication	This manuscript does not contain experimental findings.
Randomization	We used the technique of Mendelian randomization, which relies on random mating and the random assortment of alleles during meiosis to make causal inferences.
Blinding	This manuscript does not contain primary data, only secondary data from published genome-wide association studies.

Reporting for specific materials, systems and methods

We require information from authors about some types of materials, experimental systems and methods used in many studies. Here, indicate whether each material, system or method listed is relevant to your study. If you are not sure if a list item applies to your research, read the appropriate section before selecting a response.

Materials & experimental systems

n/a	Involved in the study	
☒	☐	Antibodies
☒	☐	Eukaryotic cell lines
☒	☐	Palaeontology
☒	☐	Animals and other organisms
☐	☒	Human research participants
☒	☐	Clinical data

Methods

n/a	Involved in the study	
☒	☐	ChIP-seq
☒	☐	Flow cytometry
☒	☐	MRI-based neuroimaging

Human research participants

Policy information about studies involving human research participants

Population characteristics	This manuscript does not contain primary data, only secondary data from published genome-wide association studies. Associations with HDL-cholesterol were estimated in the Global Lipids Genetic Consortium (GLGC) on up to 188,577 individuals of European ancestry, and associations with coronary heart disease (CHD) risk from the CARDIoGRAMplusC4D consortium on up to 60,801 CHD cases and 123,504 controls predominantly of European ancestry.
Recruitment	This manuscript does not contain primary data, only secondary data from published genome-wide association studies. The Global Lipids Genetic Consortium (GLGC) and CARDIoGRAMplusC4D consortium both include over 30 studies. Information about recruitment to each constituent study can be found in the manuscript describing that particular study.
Ethics oversight	This manuscript only contains analysis of anonymized publicly-available data, and so no ethics approval is required.

Note that full information on the approval of the study protocol must also be provided in the manuscript.
